# Bioactive Mushroom Polysaccharides: A Review on Monosaccharide Composition, Biosynthesis and Regulation

**DOI:** 10.3390/molecules22060955

**Published:** 2017-06-13

**Authors:** Qiong Wang, Feng Wang, Zhenghong Xu, Zhongyang Ding

**Affiliations:** 1 Key Laboratory of Carbohydrate Chemistry and Biotechnology, Ministry of Education, School of Biotechnology, Jiangnan University, Wuxi 214122, China; ldwq_86@163.com (Q.W.); zhenghxu@jiangnan.edu.cn (Z.X.); 2 School of Food and Biological Engineering, Jiangsu University, Zhenjiang 212013, China; fengwang@ujs.edu.cn; 3 National Engineering Laboratory for Cereal Fermentation Technology, Jiangnan University, Wuxi 214122, China

**Keywords:** mushroom polysaccharides, monosaccharide composition, bioactivity, structure-activity relationship, biosynthesis, regulation

## Abstract

Mushrooms are widely distributed around the world and are heavily consumed because of their nutritional value and medicinal properties. Polysaccharides (PSs) are an important component of mushrooms, a major factor in their bioactive properties, and have been intensively studied during the past two decades. Monosaccharide composition/combinations are important determinants of PS bioactivities. This review summarizes: (i) monosaccharide composition/combinations in various mushroom PSs, and their relationships with PS bioactivities; (ii) possible biosynthetic pathways of mushroom PSs and effects of key enzymes on monosaccharide composition; (iii) regulation strategies in PS biosynthesis, and prospects for controllable biosynthesis of PSs with enhanced bioactivities.

## 1. Introduction

Mushrooms, belonging to the class Basidiomycetes and sometimes Ascomycetes, are a widely distributed food resource on earth and have been consumed because of their nutritional value and medicinal properties for over 2000 years. In addition to their enjoyable flavor and taste, human health was improved by mushrooms due to their nutrients, including digestible proteins, carbohydrates, fiber, vitamins, minerals, and antioxidants [1,2]. A wide variety of bioactive compounds from medicinal mushrooms, which are widely used in eastern Asia, have been studied extensively, and these compounds including polysaccharides, lectins, lactones, terpenoids, and alkaloids have been reviewed [1,3,4]. Among these bioactive compounds, polysaccharides (PSs) with various activities are the main component for the bioactivities of some mushroom species. The diverse activities displayed by PSs include antitumor, immunomodulatory, anti-inflammatory, antinociceptive, antiviral, antioxidative, hypoglycemic, and hepatoprotective effects, as well as protection against chronic radiation stress [1,2,3,4,5].

PSs are divided into two distinct categories, homopolysaccharides (homo-PSs) and heteropolysaccharides (hetero-PSs), on the basis of monosaccharide composition. Both homo-PSs and hetero-PSs may possess homolinkages or heterolinkages in regard to configuration and/or linkage position. In addition to their differing types and sequences of monosaccharide units, hetero-PSs have various types and sequences of glycosidic linkages, resulting in practically limitless structural diversity [5]. Earlier studies suggested that homo-PSs, particularly glucans, are the only PSs with medicinal properties. However, hetero-PSs also display important biological activities [1], and both homo-PSs and hetero-PSs bioactivities are closely related to their specific structural characteristics [5]. The detailed mechanisms of bioactivities related to structure in hetero-PSs remain largely unexplored, although the essential roles of glucans and glycans have been well established. PS composition and combination are clearly correlated with pharmaceutical activities, and this relationship has been the subject of increasing research attention [6,7,8].

The production of chemically and biologically uniform PSs requires knowledge of PS synthetic pathways and regulatory mechanisms. The biosynthetic pathways of PS involve synthesis of nucleotide sugar precursors, assembly of repeating monosaccharide units, and the polymerization process. In PS biosynthetic pathways, enzymes involved in the biosynthetic pathways of nucleotide sugar precursors are closely related with production of mushroom PSs [9,10,11,12]. In submerged culture of *Ganoderma lucidum* (lingzhi mushroom), overexpression of homologous α-phosphoglucomutase and uridine diphosphate glucose pyrophosphorylase (UDP-glucose pyrophosphorylase) genes enhanced PS production [13,14]. Relationships between the monosaccharide composition of PSs and activities of related enzymes were recently described [15,16].

Numerous studies have addressed the structures and bioactivities of mushroom saccharides, and the relationship between them. Here, we review recent work in this field, with focus on the relationship between monosaccharide composition and the activities of mushroom PSs. We discuss: (i) monosaccharide composition/combinations in various mushroom PSs, and their relationships with bioactivities; (ii) possible biosynthetic pathways of mushroom PSs and effects of key pathway enzymes on monosaccharide composition; (iii) regulation strategies in PS biosynthesis, and prospects for controllable synthesis of PSs with enhanced bioactivities.

## 2. Monosaccharide Composition, Combinations, and Bioactivities of Mushroom Polysaccharides

Glucan is a well-studied PS and can be produced by different fungi with various terms, such as lentinan, ganoderan, grifolan, and schizophyllan [2,17]. Many studies have addressed the structure–bioactivity relationships of glucans, particularly lentinan [2]. Aside from glucans, no other homo-PSs have been reported. Many mushroom hetero-PSs have been reported in the past two decades, and attempts have been made to elucidate their structure–bioactivity relationships. Few of the PSs have been commercialized, apart from high production or purification costs and low or erratic PS yields, partly because of their unstable chemical characteristics, e.g., monosaccharide composition/combinations that give rise to great variability [17]. The most extensively studied monosaccharide compositions involve glucose (Glc), mannose (Man), galactose (Gal), xylose (Xyl), arabinose (Ara), rhamnose (Rha), and fucose (Fuc). Less frequently encountered components of mushroom PSs include fructose (Fru), ribose (Rib), glucuronic acid (GlcA), galacturonic acid (GalA), *N*-acetyl-glucosamine (GlcNac, GlcN), and *N*-acetyl-galactosamine (GalNac, GalN).

### 2.1. Monosaccharide Composition and Combinations

Monosaccharide compositions of PSs produced by Ascomycetes differ from those of PSs produced by Basidiomycetes. In Ascomycetes, the PSs are mainly hetero-PSs, and Glc, Man, Gal are often presented (Table 1). In a study of *Cordyceps* (*Ophiocordyceps*) *sinensis* PSs, Yan et al. reviewed that monosaccharide composition usually consisted of Glc, Man, and Gal in various molar ratios [4], and the latest research of *Cordyceps sinensis* also found the similar result [18,19]. In contrast, intracellular PSs (IPSs) from mycelia contain various polyglucans as well as Glc [20,21]. Compositions of other *Cordyceps* species were generally similar to those of *C. sinensis* [22,23,24,25], although some other monosaccharides, such as Rha, Xyl, Fru and Ara, were present in *C. cicadae* and *C. kyushuensis* [26,27]. In *Morchella esculenta*, another well-known ascomycete, the PSs contain four or more monosaccharides [28,29,30]. Zhao et al. isolated 52 PSs from fermentation systems and fruiting bodies of *Tuber* species, and found that they were all composed mainly of Man, Glc, and Gal, suggesting that *Tuber* PSs have identical chemical compositions [31]. On the other hand, two fractions of *T. huidongense* PSs were extracted from fruiting bodies, and Glc was the only monosaccharide detected in THWP-2 [32]. In *T. rufum* PS, Fuc was present in addition to Glc and Gal [33].

In comparison with Ascomycetes, PSs of Basidiomycetes are more complex, particularly in regard to monosaccharide composition and molar ratios of hetero-PSs. Many reviews have focused on chemical characterization of PSs from Basidiomycetes [1,2,3]. However, current knowledge of monosaccharide composition/combinations is not systematic. Table 2 summarizes results of studies during the past few decades of PSs from *Ganoderma* species, including monosaccharide compositions and molar ratios. The monosaccharide composition of PS from fruiting bodies, mycelia, or fermentation broth of a same fungal strain showed different. To facilitate interpretation of PS properties, Ruthes et al. have reviewed different PSs separately according to the main chain, and found most of the main chains consist solely of Glc, Man, or Gal; however, some PSs contain multiple main chains composed of two or more different monosaccharides in varying combinations, making description difficult [5].

### 2.2. Bioactivities of Mushroom PSs with Differing Monosaccharide Composition/Combinations

PSs are highly complex molecules, and detailed characterization of specific glycosidic linkages, degree of branching, monosaccharide composition, molecular weight, and chain conformation is essential for elucidating structure—biological activity relationships. Increasing attention has been paid during the past decade to the relationship between PS composition and biological activity. PSs with differing monosaccharide compositions differ in their biological activities and therapeutic effects. In some cases, PSs with high content/ratio monosaccharide, such as Man, Rha, and Fuc, appear to be responsible for the bioactivity. For examples, Man-rich exopolysaccharide (EPS) from *Tremella mesenterica* [47] and Rha-rich EPS from *Lactobacillus rhamnosus* [48] stimulate the immune system through receptors located on macrophage. l-Fuc-enriched EPS is used as a skin moisturizing agent in the cosmetic industry because l-Fuc displays anticancer and anti-inflammatory activities [49]. PSs containing high levels of uronic acid display significant antioxidant activity, and may be attributed to the presence of the functional group -COOH [7]. Functional groups that act as efficient electron or hydrogen donors are associated to the antioxidant activity of certain PSs [50].

Man is a typical component of many bioactive PSs. Most of these PSs also confer virulence against pathogenic species. Human cells are also able to recognize such carbohydrates through Man receptors, thus stimulating cytokine production [51,52]. The high molar proportion of Man in *Inonotus obliquus* PSs may enhance antioxidant activity [53,54]. Kim et al. reported an anti-cancer effect by endo-PS fractions from cultivated mycelia of *I. obliquus* with Man as the major component [55]. Chen et al. showed that the Man component of *Tremella mesenterica* EPS stimulated nitric oxide (NO) and cytokine (IL-6 and TNF-α) production in RAW 264.7 macrophage cells [47]. The major pharmacologically-active substance in *Tremella* is the PS glucuronoxylomannan, composed primarily of Man [56]. Biological activity of certain mushroom PSs is related to content of other monosaccharides such as rhamnose (Table 3). Rha as a major monosaccharide component may enhance antioxidant capacity, and play a role in radical-scavenging ability [34,57]. Biological activities displayed by active components in mushroom PSs are summarized in Table 3.

Many researchers have used a model, based on relationships between monosaccharide composition ratios and biological activities, to provide theoretical support for applications of PS from natural products. Lo et al. observed a strong correlation between monosaccharide composition and ferrous ion chelating ability, and showed that Rha level in PSs was the dominant component in modulation of the chelating response variable [6]. In another study, Lo et al. used statistical methods (multiple linear regression analysis, principal component analysis (PCA), factor analysis) to examine relationships between monosaccharide composition ratios and macrophage stimulatory activities of regionally different strains of *Lentinula edodes* [62]. Ara, Xyl, Man, and Gal were most strongly related to macrophage stimulatory activities. When additional PCA and factor analysis methods were applied to the same monosaccharide composition ratio data, Ara, Xyl, Man, and Gal compositions were again found to be the most important. Glc, although present as a major monosaccharide component in all strains and presumably forming the backbone of PS structures, was not a major determinant factor for structural characteristics or *in vitro* macrophage stimulatory activities [62]. Li et al. used multiple linear regression (MLR), support vector machine (SVM), and artificial neural network (ANN) methods to establish an optimum quantitative structure–activity relationship (QSAR) model for the antioxidant activity of PSs. In MLR and ANN models, Ara and GalA played major roles in determining EC_50_ of DPPH (1,1-Diphenyl-2-picrylhydrazyl)-scavenging activity, and Fuc, Rha, and uronic acid had the greatest effects on the hydroxyl radical scavenging activity of PSs. Such measurement methods are useful for predicting performance and evaluating relationships between PS properties and biological activities [7].

Monosaccharide composition/combinations clearly play an important role, and are strongly correlated with biological activity. Chemically and biologically uniform PSs with specific monosaccharide composition/combinations could be used for controllable biosynthesis. However, we know little about the detailed mechanisms of the bioactive effects relating to the monosaccharide composition of PS, although the bioactivities of PSs are well established.

## 3. Biosynthetic Pathways and Key Enzymes of Mushroom Polysaccharides

### 3.1. Biosynthetic Pathways

Fungal PSs are involved in a wide variety of cellular functions, including energy storage, cell wall structure, cell–cell interactions and signaling, host–pathogen interaction, and protein glycosylation [5]. The PSs are synthesized using intracellular nucleotide sugars at the membrane level to construct the main structure of cell walls or form a gel-like matrix on the hyphal surface. In submerged fermentation, gel-like PSs may be further excreted into the growth medium and are referred to as EPSs [63]. Currently, the biosynthetic pathways of mushroom PSs remain unclear because of the inadequate knowledge of relative enzymes and its functions in pathway, which should be proved by gene cloning and genetic transformation.

**Figure 1:**
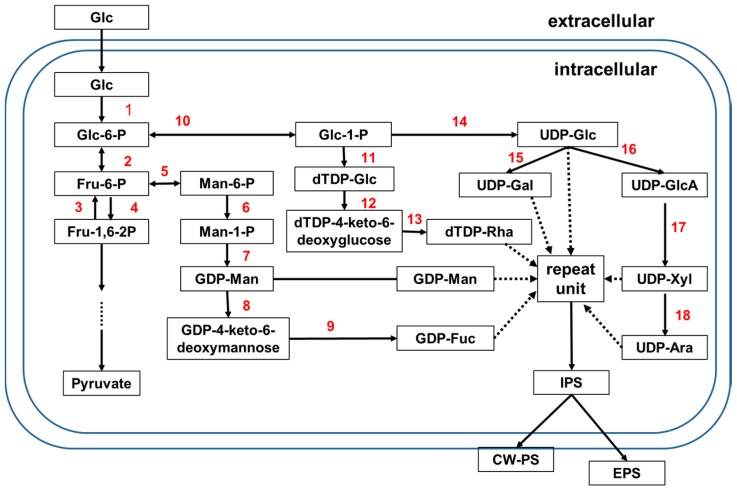
Proposed/deduced/hypothesized biosynthetic pathways for mushroom PSs. 1. Glucokinase (GK); 2. Phosphoglucose isomerase (PGI); 3. Fructose-1,6-biphosphatase (FBPase); 4. Phosphofructokinase (FPK); 5. Phosphomannose isomerase (PMI); 6. Phosphomannose mutase (PMM); 7. GDP-Man pyrophosphorylase (GMP); 8. GDP-Man dehydratase (GMD); 9. GDP-4-keto-6-deoxymannose epimerase/reductase (GMER); 10. Phosphoglucose mutase (PGM); 11. dTDP-glucose pyrophosphorlase (dTDP-GPP); 12 and 13. dTDP-Rha synthase (dTRS); 14. UDP-Glc pyrophosphorylase (UGP); 15. UDP-Gal-4-epimerase (UGE); 16. UDP-Glc dehydrogenase (UGDG). 17. UDP-glucuronate decarboxylase/UDP-Xyl synthase (UXS); 18. UDP-Xyl-4-epimerase (UXE).

However, a simplified biosynthetic pathway of mushroom PSs has been constructed based on identification of intermediate compounds and activities of synthesis-related enzymes by various researchers [11,16,64]. Approaches such as gene expression [13,14] and RNAi-mediated gene silencing [64] have also been applied for study of the biosynthetic pathway. In Figure 1, we propose a more detailed biosynthetic pathway based on the previous publications. In this model, the biosynthetic pathway of nucleotide sugar precursors in mushroom PSs is similar to that in plants [65], providing a basis for analysis of other metabolic pathways in mushroom PSs. The genomes of *Ganoderma lucidum* [66], *Antrodia cinnamomea* [67], *Lentinula edodes* [68], and *Lignosus rhinocerotis* [69] have been published. These genomic studies provide insights into the genetic basis of reported medicinal properties of PSs, and a platform for further characterization of putative bioactive PSs and pathway enzymes.

### 3.2. Effects of Key Enzymes on PS Production and Monosaccharide Composition

Enzymes in the PS synthesis pathways of mushrooms play an important role in PS production and regulation of monosaccharide composition. These enzymes include Phosphoglucose isomerase, α-phosphoglucomutase, UDP-Glc pyrophosphorylase and so on. Phosphoglucose isomerase (PGI; EC 5.3.1.9) catalyzes the interconversion of Glc-6-phosphate and Fru-6-phosphate, and plays key roles in glycolysis and gluconeogenesis pathways. PGI catalyzes the reaction that generates Fru-6-phosphate, the precursor of GDP-Man in PS synthesis [16]. Thus, PGI activity level determines the direction of Glc-6-phosphate conversion. PGI may also control PS synthesis to some extent. Zhu et al. observed a strong correlation between PGI activity level and the amount of IPS produced [11]. Yang et al. showed that the addition of coix lacryma-jobi oil (CLO) to culture medium enhanced EPS and IPS production by directly affecting synthesis levels of PGI and α-phosphoglucomutase (PGM) at different stages [70]. In a study by Wei et al., higher sucrose level increased PGM activity, and also enhanced PS production through inhibition of PGI activity [12]. Similar results were reported by Tang and Zhong [71].

α-phosphoglucomutase (PGM; EC 5.4.2.2), a key enzyme in carbohydrate metabolism, catalyzes the reversible transfer of a phosphate group between C-1 and C-6. PGM converts Glc-6-P and Glc-1-P; Glc-6-phosphate enters a glycolysis pathway to yield energy, and Glc-1-phosphate is the precursor of sugar nucleotides (e.g., UDP-Glc, dTDP-Rha, UDP-Gal) in PSs [10,16]. In the study by Tang and Zhong, PGM activity was correlated with the amount of EPS produced by *G. lucidum* [71]. Xu et al. investigated the effects of PGM gene overexpression on PS production and transcription levels of three genes, and demonstrated that the PGM gene is an important regulatory gene in PS biosynthesis in *G. lucidum* [14]. PGM gene overexpression in submerged culture resulted in increased PS production and upregulation of downstream genes encoding the enzymes involved in PS biosynthesis, including PGM, UDP-Glc pyrophosphorylase (UGP), and β-1,3-glucan synthase (GLS) [14]. PGM activity was also affected by the addition of coixenolide during PS production in *G. lucidum* [10]. In a submerged culture of *C. militaris*, PGM was the key enzyme for regulation of IPS biosynthesis [11].

UDP-Glc pyrophosphorylase (UGP; also known as UGPase or UTP: Glc-1-phosphate uridylyltransferase; EC 2.7.7.9), a member of the Pfam glycosyl transferase family (PF01702), catalyzes the reversible interconversion of Glc-1-phosphate and UDP-Glc [64]. UGP is a key enzyme involved in carbohydrate metabolism and cell wall biosynthesis [72]. Studies by Liao et al. and Jiang et al. showed that the UGP gene (*ugp*) is associated with the synthesis of LPS and EPS surface structures, because its substrate/product UDP-Glc is the precursor of these PSs [73,74]. Li et al. cloned the *ugp* gene from *G. lucidum*, and examined the effect of RNAi silencing of *ugp* on PS synthesis. In liquid culture, EPS concentration in *ugp*-silenced strains was much lower (30–56%) than that in wild type (WT) strains. In contrast, IPS concentration did not differ significantly between *ugp*-silenced and WT strains [64]. Ji et al. showed that overexpression of homologous *ugp* in submerged culture *G. lucidum* enhanced PS production; maximal IPS and EPS levels in the *ugp*-overexpressing strains were respectively 42% and 36% higher than in WT strains [13]. Zhu et al. found that higher UGP activity was correlated with higher IPS biosynthesis rate in *Cordyceps militaris* [11], suggesting an important role of UGP in PS biosynthesis. However, a controversial conclusion was found by Zhou et al., Wei et al., and Yang et al.: that there was no significant association of UGP activity with PS biosynthesis [10,12,70], which may be attributed to the different strains and methods. Therefore, the exact reason still requires further research.

Enzyme activities of glucokinase (GK) [16], UDPG-dehydrogenase (UGDG) [11], and dTDP-Glc pyrophosphorylase (dTDP-GPP) [10,70] have also been investigated. However, these studies showed no notable association between PS biosynthesis and activities of these enzymes.

**Figure 2:**
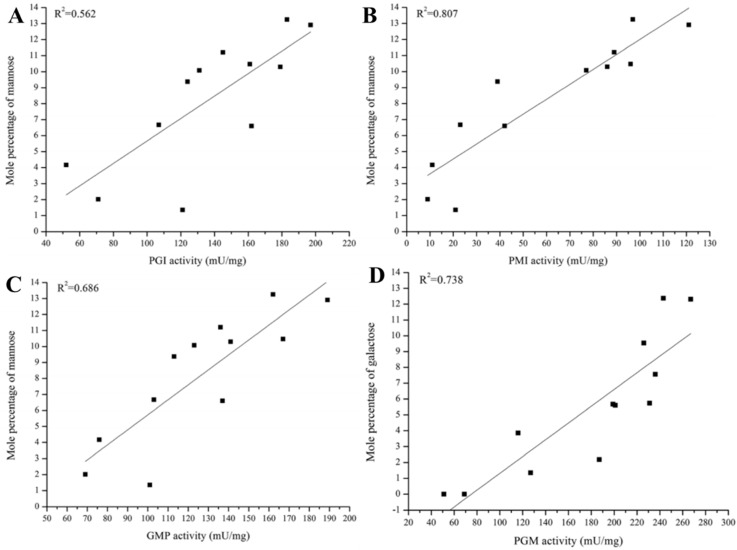
Correlations (*R*^2^) between activities of exopolysaccharide (EPS) synthesis enzymes and monosaccharide mole percentages under various carbon source conditions. (**A**) Phosphoglucose isomerase (PGI) and Man; (**B**) Phosphomannose isomerase (PMI) and Man; (**C**) GDP-Man pyrophosphorylase (GMP) and Man; (**D**) Phosphoglucose mutase (PGM) and Gal.

Relationships between the monosaccharide composition of PS and the activity of related enzymes were recently studied using a correlation coefficient (*R*^2^) based on data obtained under various culture and carbon source conditions [15,16]. Changes in PGM, PGI, and phosphomannose isomerase (PMI) activities were strongly correlated with Gal and Man mole percentages in *G. lucidum* EPS, based on *R*^2^ values obtained under various culture temperatures and initial pH values [15]. In contrast, under various carbon source conditions, PGI and PMI activities were correlated only with Man mole percentage, and PGM activity was correlated only with Gal mole percentage (Figure 2) [16]. GDP-Man pyrophosphorylase (GMP) activity, whose *R*^2^ with mole percentages of all monosaccharides under various culture conditions was low (<0.5), showed a stronger correlation with Man mole percentage under carbon source conditions. Taken together, results of these studies indicate that PGM, PGI, and PMI regulate the mole percentages of Gal and Man in EPS under various culture and carbon source conditions, whereas GMP regulates the mole percentage of Man only in response to carbon source. Key enzymes regulating monosaccharide synthetic pathways in *G. lucidum* EPS can thus be divided into two groups, based on responses to culture conditions and culture medium. Mole percentages of other monosaccharides (Glc, Ara, Fuc) and activities of other tested enzymes under the various conditions showed no significant correlations (*R*^2^ < 0.5) [16].

## 4. Factors Regulating Mushroom Polysaccharide Synthesis

Monosaccharide composition/combinations significantly affect PS bioactivity. Increasing research attention has been paid to regulation of synthesis of PS with stronger bioactivities. Variability in monosaccharide composition/combinations among mushroom PSs may result from strain variations, developmental stage, culture method and conditions, medium composition, extraction method, and even drying method [60,75,76,77,78,79]. In industrial-scale operations, submerged fermentation procedures have been successfully applied for mushroom cultivation during the past two decades. Submerged culture has advantages over traditional solid-state culture in terms of higher mycelial biomass production of bioactive metabolites in a compact space, shorter times, and reduced contamination [75,80]. This is a useful alternative approach for efficient production of IPS and EPS.

Structural parameters (monosaccharide composition, degree of branching, molecular weight, molecular weight distribution, chain conformation) are affected by the fermentation medium [81,82]. Among the various nutrients present in the culture medium, carbon source is an important factor determining the monosaccharide content of PS. In a study by Peng et al., the use of sugar as a carbon source was associated with an excess of the same monosaccharide in *G. lucidum* EPS. With mixed carbon sources, the main containing monosaccharides of *G. lucidum* EPS were Glc, Gal, and Man, and the combined mole percentage of EPS monosaccharides was the same as carbon sources used in culture medium. [16]. In *Pleurotus pulmonarius* submerged culture, each EPS sample had Man, Gal, and Glc as major monosaccharide components. Water-insoluble polysaccharides (IEPS) fractions obtained from Glc- and Xyl-based culture media (termed IEPS-Glc and IEPS-Xyl, respectively) contained Glc as the sole monosaccharide component. Surprisingly, these glucans were not isolated from Gal- and Ara-based culture media [83]. In *Paecilomyces hepiali*, a high percentage of Man in EPS was observed under Glc carbon source, and a high percentage of Glc was observed under Man carbon source [63]. These findings indicate that monosaccharide composition and mole percentages of PS are determined by carbon source in mushroom submerged culture. In a study of *Cordyceps sinensis* CS-HK1 mycelial cultures, the monosaccharide composition of EPS isolated on different days varied according to carbon source. The monosaccharides Glc, Gal, and Man were found in all EPS samples, with Glc being the major component (50–75% of total). The addition of Gal or Man to the culture medium resulted in no notable increase (in some cases, even a slight decrease) in their content in EPS [84].

In a study of *Tuber melanosporum* by Zhao et al., the addition of metal ions to culture medium affected IPS and EPS biosynthetic pathways. Gal, Glc, and Man were the major monosaccharides in both IPS and EPS. The addition of Mg^2+^ resulted in a maximal Man content (27.6%) in IPS, but no significant change in Gal or Glc content in IPS or EPS. Both IPS and EPS were acidic hetero-PSs containing Man, Gal, and Glc [81]. The metal ions may be a cofactor in enzymatic reactions in the PS biosynthesis pathway and alter some key enzyme activities, which lead to different molar ratios of the monosaccharide components [81]. Alternatively, addition of metal ions is an effective method to regulate the structural properties of polysaccharide.

## 5. Conclusions and Future Perspectives

A steadily increasing number of PSs have been extracted from a wide variety of mushroom species during the past decade. In many cases, their structural features and bioactivities have been elucidated through the application of advanced analytical techniques. However, there is no uniformity or predictability in the structural features or functional characteristics of bioactive PSs. The bioactivity mechanisms, biosynthetic pathways, and productivities of the PSs are also highly variable and confusing to researchers. Monosaccharide composition/combinations is an important characteristic of PSs, and there is a clear relationship between structure and bioactivity. Continued investigation along this line will help to identify the most effective structures, and lead to improved regulation strategies in PS biosynthesis and prospects for the controllable synthesis of PSs with enhanced bioactivities.

## Figures and Tables

**Table 1:** Monosaccharide composition and molar ratio of polysaccharides from Ascomycetes.

Species	Source	Monosaccharide Composition	Molar Ratio	Reference
*Cordyceps sinensis*	mycelium	Man:Glc:Gal:GlcA	9.6:4.0:4.4:1.0	[19]
*Cordyceps militaris*	fruiting body	Man:Gal:Glc	56.7:34.5:8.8	[22]
	NA	Man:Glc:Gal	2.84:1:1.29	[23]
		Man:Glc:Gal	2.05:1:1.09	
	fruiting body	Man:Glc:Gal	1:28.63:1.41	[24]
		Man:Glc:Gal	1:12.41:0.74	
*Cordyceps sobolifera*	fruiting body	Man:Glc:Gal	1.7:8.9:1	[25]
*Cordyceps cicadae*	NA	Glc:Rha:Xyl:Man:Ara:Gal	63.10:39.11:20.12:15.16:2.05:0.12	[26]
*Cordyceps kyushuensis*	stroma	Fru:Man:Rha:GalN:Ara	1.0:1.19:0.11:0.11:0.34	[27]
		Fru:Man:Rha:Glc:Ara	1:1.29:0.14:0.07:0.32	
*Morchella esculenta*	fruiting body	Xyl:Glc:Man:Rha:Gal	5.4:5.0:6.5:7.8:72.3	[28]
	fruiting body	Glc:Man:Gal:Ara:Xyl:Rha	38.06:14.43:17.06:9.25:2.08:0.94	[29]
		Glc:Man:Gal:Ara:Xyl:Rha	27.04:28.66:11.12:9.07:6.71:3.22	
		Glc:Man:Gal:Ara:Xyl:Rha	24.69:20.46:10.22:7.91:4.05:2.83	
	fermentation broth	Ara:Man:Glc:Gal	0.7:2.8:24.8:1.0	[30]
		Rha:Man:Glc:Gal	1.8:3.1:21.4:1.0	
*Tuber huidongense*	fruiting body	Glc:Man:Gal	60.56:20.12:19.32	[32]
		Glc	-	
*Tuber rufum*	fruiting body	Glc:Gal:Fuc	4:3:1	[33]

Glc: glucose; Man: mannose; Gal: galactose, Xyl: xylose; Ara: arabinose, Rha: rhamnose, Fuc: fucose; GlcA: glucuronic acid; GalN: *N*-acetyl-galactosamine.

**Table 2:** Monosaccharide composition and molar ratio of polysaccharides from *Ganoderma* species.

Species	Source	Monosaccharide Composition	Molar Ratio	Reference
*Ganoderma lucidum*	fruiting body	Rha:Ara:Xyl:Man:Glc:Gal	11.4:30:27.1:8:9.1:14.3	[34]
		Rha:Ara:Xyl:Glc:Gal	44.7:20.9:19.9:3.6:10.8	
	NA	Man:Rha:Glc:Gal	4.7:0.65:65.22:29.43	[35]
	fruiting body	Glc	-	[36]
		Glc:Gal:Man	29:1.8:1.0	
	fruiting body	Glc	-	[37]
	fermentation broth	Gal:Man:Glc:Ara:Rha	103:17:12:10:3	[38]
	fruiting body	Glc:Fuc:Gal	1.0:1.09:4.09	[39]
	fruiting body	Gal:Rha:Glc	1.00:1.15:3.22	[40]
	fermented soybean curd residue	Ara:Rha:Xyl:Man:Glc	4.66:1.23:3.1:0.61:1.29	[41]
		Ara:Xyl:Glc	2.82:1.33:0.87	
		Ara:Rha:Xyl:Gal:Man:Glc	5.09:0.52:1.07:1.29:0.48:2.76	
*Ganoderma atrum*	fruiting body	Gal:Fuc:Glc	75.87:11.83:6.02	[42]
	fruiting body	Glc:Man:Gal:GalA	4.91:1:1.28:0.71	[43]
*Ganoderma sinense*	fruiting body	Man:Glc:Gal	4.7:27.1:1.0	[44]
*Ganoderma tsugae*	mycelium	Fuc:Xyl:Man:Gal:Glc:GlcNac	2.9:16.1:66.7:9.4:0.1:4.3	[45]
		Fuc:Xyl:Man:Gal:Glc:GlcNac	4.5:1.3:58.0:24.5:3.8:7.9	
*Ganoderma formosanum*	fruiting body	Man:Gal:Glc:Ara:Fuc:Fru:Rha	50.13:13.1:17.47:6.94:2.71:9.21:0.45	[46]
		Man:Gal:Glc:Ara:Fuc:Rha	44.91:38.64:8.26:0.08:8.02:0.09	
		Man:Gal:Glc:Ara:Fuc:Fru:Rha	33.35:30.84:20.52:8.78:4.44:1.33:0.74	

Glc: glucose; Man: mannose; Gal: galactose, Xyl: xylose; Ara: arabinose, Rha: rhamnose, Fuc: fucose; GalA: galacturonic acid; GlcNac: *N*-acetyl-glucosamine.

**Table 3:** Biological activities of active components in mushroom PSs.

Species	Biological Activity	Active Component	Reference
*Inonotus obliquus*	antioxidant and anticancer activity	Man	[53,54,55]
*Tremella mesenterica*	cytokine-stimulating activity	Man	[47]
*Ganoderma lucidum*	antioxidant activity	Rha	[34]
*Flammulina velutipes*	antioxidant activity	Rha	[57]
*Agrocybe cylindracea*	antioxidant activity	Glc, Gal	[58]
*Sarcodon aspratus*	macrophage activation	Man, Rha	[59]
*Inonotus obliquus*	antioxidant activity	Man, Rha, Xyl	[8]
*Hirsutella* sp*.*	antioxidant activity	Man, Glc	[60]
*Pleurotus eryngii*	antioxidant activity	Man, Rha, GalA	[61]
